# Intracellular Desmoglein-2 cleavage sensitizes epithelial cells to apoptosis in response to pro-inflammatory cytokines

**DOI:** 10.1038/s41419-018-0380-9

**Published:** 2018-03-09

**Authors:** Mark Yulis, Miguel Quiros, Roland Hilgarth, Charles A. Parkos, Asma Nusrat

**Affiliations:** Department of Pathology, The University of Michigan, Ann Arbor, MI 48109 USA

## Abstract

Desmosomal cadherins mediate intercellular adhesion and have also been shown to regulate homeostatic signaling in epithelial cells. We have previously reported that select pro-inflammatory cytokines induce Dsg2 ectodomain cleavage and shedding from intestinal epithelial cells (IECs). Dsg2 extracellular cleaved fragments (Dsg2 ECF) function to induce paracrine pro-proliferative signaling in epithelial cells. In this study, we show that exposure of IECs to pro-inflammatory cytokines interferon-gamma (IFN-γ) and tumor necrosis factor-alpha (TNF-α) resulted in Dsg2 intracellular cleavage and generation of a ~55 kDa fragment (Dsg2 ICF). Dsg2 intracellular cleavage is mediated by caspase-8 and occurs prior to Dsg2 extracellular cleavage and the execution of apoptosis. Expression of exogenous Dsg2 ICF in model IECs resulted in increased sensitivity to apoptotic stimuli and apoptosis execution. Additionally, expression of the Dsg2 ICF repressed the anti-apoptotic Bcl-2 family member proteins Bcl-X_L_ and Mcl1. Taken together, our findings identify a novel mechanism by which pro-inflammatory mediators induce modification of Dsg2 to activate apoptosis and eliminate damaged cells, while also promoting release of Dsg2 ECF that promotes proliferation of neighboring cells and epithelial barrier recovery.

## Introduction

Intestinal epithelial cells are a critical component of the intestinal mucosal barrier. This barrier serves as an interface between distinct luminal and mucosal environments and is essential to maintaining tissue homeostasis^1^. The intestinal epithelium is highly dynamic and is actively turned over in less than a week. Yet, throughout this process, the epithelial barrier properties are maintained. Intestinal epithelial barrier compromise has been reported to contribute to the pathogenesis of mucosal inflammatory disorders such as inflammatory bowel disease^2^. Epithelial barrier function is achieved by a series of intercellular junctions that include the tight junctions, adherens junctions, and desmosomes^3,4^. Intercellular junctional proteins not only serve to control epithelial adhesion and barrier function, but also play an active role in regulating epithelial homeostasis encompassing cell proliferation, migration, and differentiation^5–8^.

Ultrastructural studies have visualized desmosomes as spot welds between intestinal epithelial cells (IECs). These junctions are located within the lateral membrane below the tight junctions and adherens junctions^3^. The basic structural components of desmosomes are the transmembrane cadherin proteins (the desmogleins and desmocollins) and intracellular plaque proteins including members of the plakin, armadillo, and catenin families among others that serve a diverse range of critical functions^9,10^. Desmosomal cadherins are essential for establishing and maintaining the adhesive properties of the desmosomes. IECs exclusively express the desmosomal cadherins desmoglein-2 (Dsg2) and desmocollin-2 (Dsc2)^9^. Previous studies have identified pro-inflammatory mediators that initiate proteolytic cadherin cleavage during mucosal inflammation^5,8^. Cadherin cleavage products have been shown to have biological properties that influence epithelial homeostatic functions and intercellular adhesion^5,8,11–16^.

We have previously shown that an intracellular fragment (ICF) of Dsg2 was generated in response to camptothecin, an intrinsic apoptotic stimulus. Dsg2 ICF generation was associated with increased IEC apoptosis^13^. Apoptosis can occur through two main pathways, intrinsic and extrinsic^17^. The intrinsic pathway is activated in response to apoptotic stimuli originating within the cell (i.e., excessive DNA damage) and is characterized by release of pro-apoptotic proteins from within the mitochondria through mitochondrial outer membrane permeabilization (MOMP). The extrinsic pathway is activated in response to stimuli originating from outside the cell (i.e., TNF-α). However, IECs are type 2 extrinsic apoptotic cells, which require MOMP for full execution of apoptosis even in response to extrinsic stimuli^18^. Therefore, regulation of mitochondrial engagement in these cells is important for the execution of apoptosis.

The Bcl-2 protein family are key regulators of mitochondrial engagement in apoptosis^18,19^. This family consists of three major groups, anti-apoptotic members (e.g., Bcl-X_L_, Bcl-2, Mcl1, etc.), pro-apoptotic members (e.g., BID, BAD, NOXA, PUMA, etc.), and effectors (BAX, BAK, and BOK)^18,19^. The anti-apoptotic members prevent MOMP either through direct interaction with the effectors or by directly interacting with pro-apoptotic family members^18,19^. Modulation of Bcl-2 protein function is primarily achieved through two types of mechanisms, (1) changing Bcl-2 protein stability/expression and/or (2) interfering with their binding^19^.

In this study, we report that the pro-inflammatory cytokines TNF-α and IFN-γ induce generation of the Dsg2 ICF. We also demonstrate that this occurs prior to Dsg2 extracellular cleavage and the execution of apoptosis. Our data show that Dsg2 intracellular cleavage is mediated by caspase-8 and also occurs in response to another extrinsic apoptotic mediator, TNF-α related apoptosis-inducing ligand (TRAIL). Using adenoviral expression vectors encoding myc-tagged Dsg2 ICF, we show that the Dsg2 ICF promotes apoptosis sensitization that is associated with downregulation of the anti-apoptotic Bcl-2 family proteins Bcl-X_L_ and Mcl1. These data indicate that pro-inflammatory cytokines promote Dsg2 intracellular cleavage, which contributes to the signaling pathways leading to epithelial apoptosis.

## Results

### TNF-α and IFN-γ promote Dsg2 intracellular cleavage

**Fig. 1:**
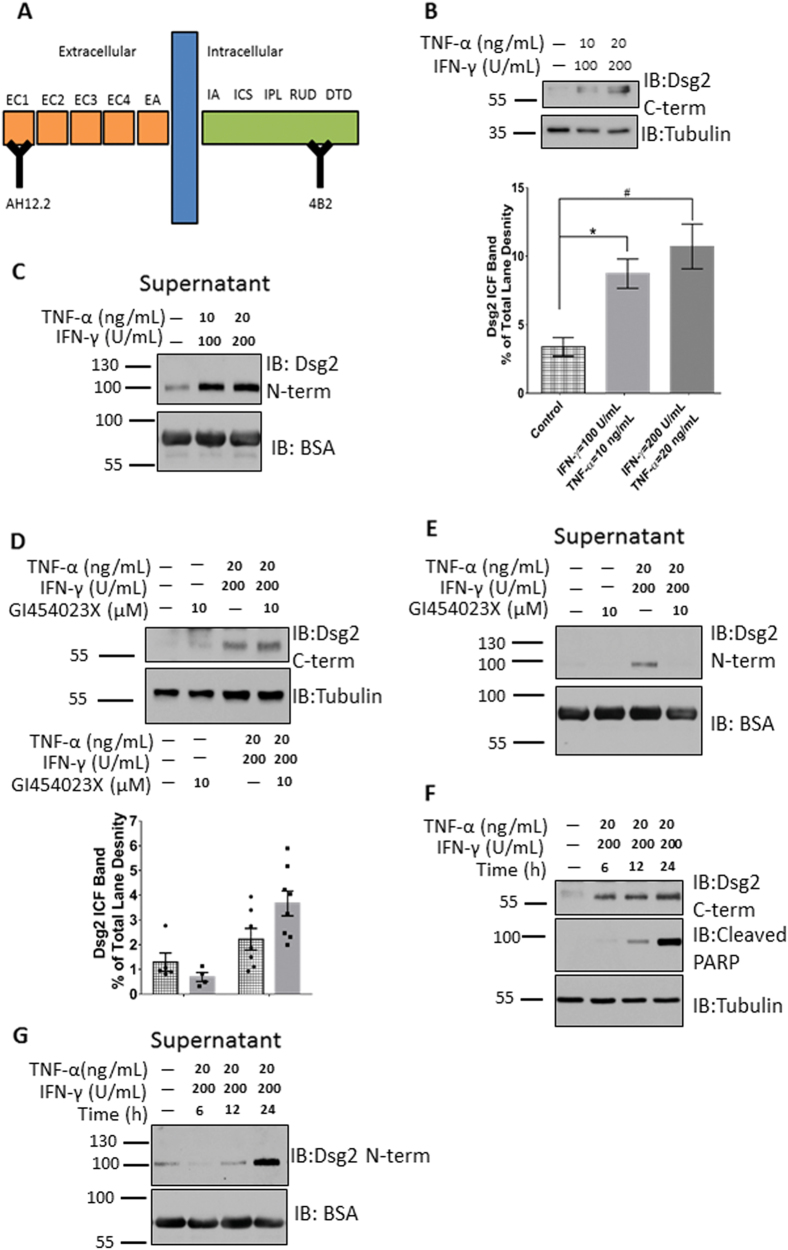
TNF-α and IFN-γ promote Dsg2 intracellular and extracellular cleavage. **a** Model of Dsg2 with mapped epitopes for AH12.2 (extracellular [N-term] specific) and 4B2 (intracellular [C-term] specific) monoclonal antibodies. EC extracellular cadherin domain, EA extracellular anchor, TM transmembrane domain, IA intracellular anchor, ICS intracellular cadherin-typical sequence, IPL intracellular proline-rich linker domain, RUD repeated unit domain, DTD desmoglein-specific terminal domain. **b** Top. Western blot using an antibody against Dsg2 C-term of cell lysates of T-84s treated with TNF-α and IFN-γ for 24 h. Bottom. Densitometry of blots. Density for all bands in each lane was collected. The ratio of the density of the ~55 kDa Dsg2 ICF band to the density of all bands for a given lane was then calculated. The data in the graph is the average of these ratios within each experimental group. *p = 0.0067; ^#^*p* = 0.0003, *n* = 11 per group. For full lanes of Dsg2 C-term blot, please see Supplemental Fig. 2A. **c** Western blot using an antibody against Dsg2 N-term of cell culture supernatants of T-84s treated as in Fig. 1b. For full lanes of Dsg2 N-term blot please, see Supplemental Fig. 2B. **d** Top. Western blot using an antibody against Dsg2 C-term of cell lysates of T-84s treated with TNF-α and IFN-γ in the presence or absence of GI254023X for 24 h. Bottom. Densitometry of blots. Densitometry collected and analyzed as for Fig. 1b. *n* ≥ 4 per group. **e** Western blot using an antibody against Dsg2 N-term of cell culture supernatants of T-84s treated as in Fig. 1d. **f** Western blot using an antibody against Dsg2 C-term of cell lysates of T-84s treated with TNF-α and IFN-γ for 6, 12, or 24 h. **g** Western blot using an antibody against Dsg2 N-term of T-84 cell culture supernatants of cells treated as in Fig. 1f. All blots are representative of at least three independent experiments

Pro-inflammatory cytokines released into the epithelial milieu during inflammation influence cellular homeostasis and barrier function^1^. We have previously reported that select pro-inflammatory cytokines induce Dsg2 ectodomain cleavage and shedding from intestinal epithelial cells^5^. Given these observations, we next determined if TNF-α and IFN-γ promote intracellular cleavage of Dsg2, influencing epithelial cell fate. TNF-α and IFN-γ dose–response studies were performed in the barrier forming model intestinal epithelial cell line T-84 (Supplemental Fig. 1). Using an epitope-mapped monoclonal antibody (4B2) that binds to a Dsg2 intracellular epitope (Fig. 1a), we observed that TNF-α and IFN-γ exposure for 24 h promoted Dsg2 intracellular cleavage with generation of a ~55 kDa Dsg2 intracellular fragment (Dsg2 ICF)^20^ (Fig. 1b). Immunofluorescence labeling and confocal microscopy using the Dsg2 C-terminal-specific antibody 4B2 revealed that TNF-α and IFN-γ exposure for 24 h led to redistribution of Dsg2 cytoplasmic tail from the plasma membrane (Supplemental Fig. 3). As we have previously reported, TNF-α and IFN-γ treatment also promoted Dsg2 ectodomain shedding into the cell culture supernatant (Fig. 1c) as detected by immunoblotting using the Dsg2 ectodomain-specific epitope-mapped monoclonal antibody, AH12.2^5^.

We next asked if there is a direct mechanistic link between Dsg2 ectodomain shedding and generation of the Dsg2 ICF. T-84 model IECs were treated with TNF-α and IFN-γ in the presence or absence of a specific ADAM10 inhibitor (GI454023X), which was previously demonstrated to prevent Dsg2 extracellular cleavage^5^. While ADAM10 inhibition influenced Dsg2 ectodomain shedding, this treatment did not impact Dsg2 ICF generation in response to TNF-α and IFN-γ treatment (Fig. 1d, e). To characterize the kinetics of Dsg2 cleavage, time-course experiments of TNF-α and IFN-γ treatment were performed. Dsg2 ICF was generated within 6 h of TNF-α and IFN-γ treatment, which is prior to Dsg2 ECF cleavage and release into the cell culture supernatant (Fig. 1f, g). Since Dsg2 ICF generation was previously associated with increased apoptosis^13^, we assayed the kinetics of PARP cleavage, which was observed 12 h after cytokine exposure (Fig. 1f). These data demonstrate that TNF-α and IFN-γ induce Dsg2 ICF cleavage, which occurs prior to Dsg2 ectodomain shedding and apoptosis execution.

### Calpain inhibition leads to accumulation of active caspase-8 and promotes Dsg2 ICF generation

**Fig. 2:**
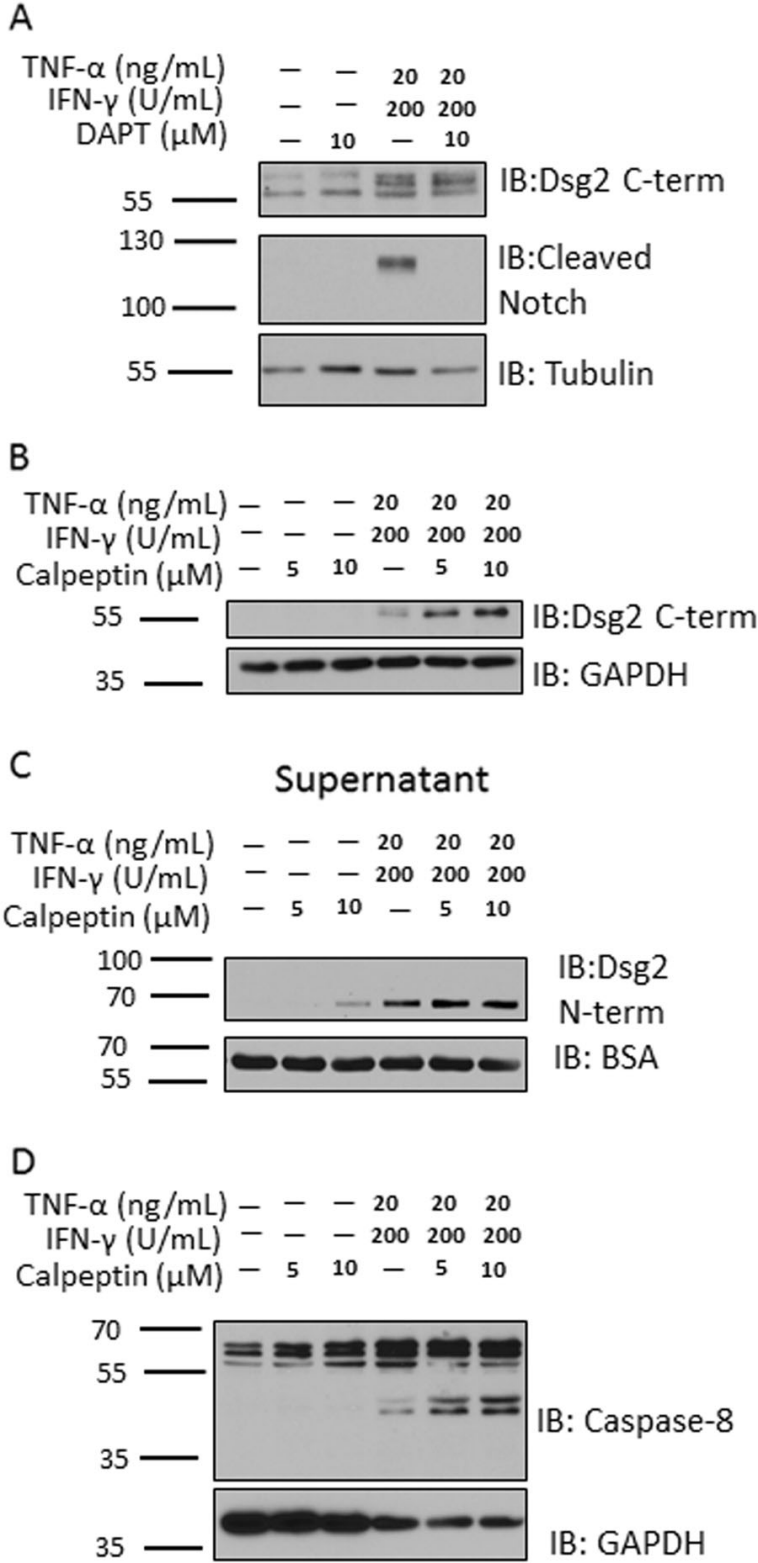
Effects of inhibition of candidate proteases on Dsg2 cleavage. **a** Western blot using an antibody against Dsg2 C-term and also for cleaved Notch of cell lysates of T-84s treated with TNF-α and IFN-γ for 24 h in the presence or absence of the γ-secretase inhibitor DAPT. **b** Western blot using an antibody against Dsg2 C-term of cell lysates of T-84s treated with TNF-α and IFN-γ in the presence or absence of increasing concentrations of calpeptin for 24 h. **c** Western blot using an antibody against Dsg2 N-term of cell culture supernatants of T-84s treated as in Fig. 2b. **d** Western blot for caspase-8 cell lysates of T-84s treated as in Fig. 2b. All blots are representative of at least three independent experiments

Pro-inflammatory cytokine signaling has been shown to activate proteases that influence cellular outcomes^5,8,21^. To examine the molecular mechanisms by which TNF-α and IFN-γ promote intracellular cleavage of Dsg2, we next explored the contribution of candidate intracellular proteases that mediate this cleavage event. Since γ-secretase is responsible for generating the intracellular C-terminal fragment-2 (CTF2) fragments of both E- and N-cadherin, we assessed the role of this protease in mediating Dsg2 cleavage^22,23^. However, inhibition of γ-secretase with DAPT (N-[N-(3,5-Difluorophenacetyl)-l-alanyl]-S-phenylglycine t-butyl ester) did not affect Dsg2 intracellular cleavage in response to TNF-α and IFN-γ (Fig. 2a).

The calpain family of intracellular cysteine proteases have been previously implicated in cadherin cleavage^24^. To test the potential role of calpains in the generation of the Dsg2 ICF, we treated IECs with TNF-α and IFN-γ in the presence and absence of the calpain inhibitor, calpeptin. In the presence of these cytokines, calpeptin treatment resulted in increased generation of the Dsg2 ICF as well as Dsg2 ectodomain shedding (Fig. 2b, c). Another family of intracellular proteases shown to have a role in cadherin cleavage are the caspases^14,22^. Calpains have been shown to inhibit the activity of caspases-8 and -9 through cleavage of the active form of these caspases^25^. Caspase activity was modulated by inhibition of calpains following TNF-α and IFN-γ treatment as evidenced by an increased abundance of active caspase-8 (Fig. 2d). These data suggest that calpain inhibition enhances the generation of both the Dsg2 ICF and ECF and this effect may be mediated by alleviation of calpain-induced cleavage of caspases.

### Caspase-8 is responsible for generating the Dsg2 ICF

**Fig. 3:**
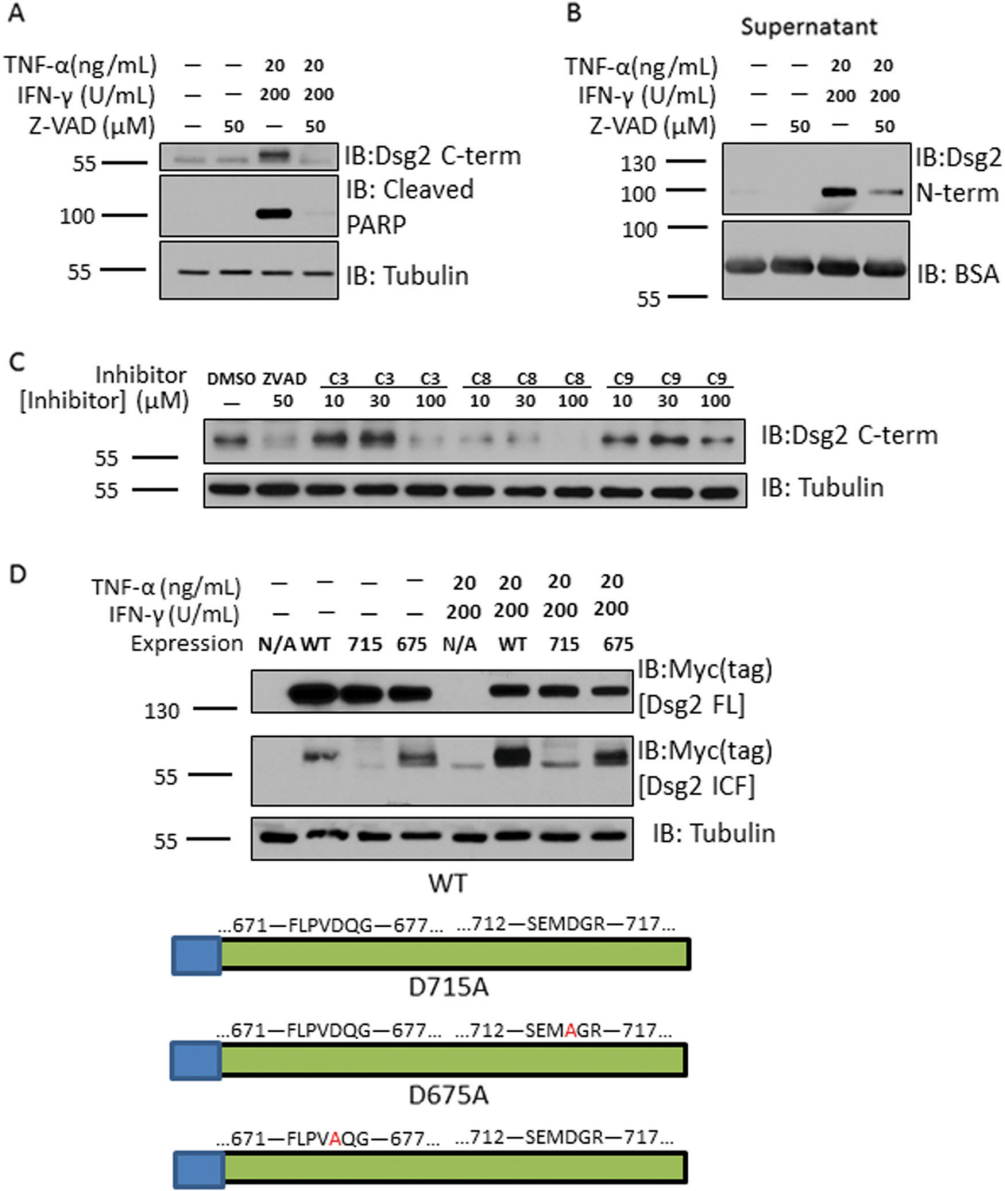
Caspase-8 is responsible for Dsg2 ICF generation. **a** Western blot using an antibody against Dsg2 C-term of cell lysates of T-84s treated with TNF-α and IFN-γ in the presence or absence of Z-VAD-fmk for 24 h. **b** Western blot using an antibody against Dsg2 N-term of cell culture supernatants of T-84s treated as in Fig. 3a. **c** Western blot using an antibody against Dsg2 C-term of cell lysates of T-84s treated with TNF-α and IFN-γ in the presence or absence of increasing concentrations of either Z-DEVD-fmk, Z-IETD-fmk, or Z-LEHD-fmk for 24 h. **d** Cos7 cells were transfected with expression plasmids encoding either WT Dsg2 [WT], or Dsg2 containing one of two putative caspase-8 cleavage consensus site mutations (D715A [715] or D675A [675]). Cells treated with Lipofectamine 3000 reagents alone with no DNA were used as controls [N/A]. Top. Western blot against the Myc tag (Top = Dsg2 full-length band; Middle = Dsg2 ICF band; Bottom = Tubulin). Bottom. Model of WT Dsg2, D715A, and D675A depicting region of Dsg2 containing sequences mutated in each highlighting the mutated residues in red. All blots are representative of at least three independent experiments

To further determine the role of caspases in mediating Dsg2 cleavage, T-84 model IECs were incubated with TNF-α and IFN-γ in the presence and absence of the pan-caspase inhibitor, Z-VAD-fmk. Pan-caspase inhibition resulted in complete loss of the Dsg2 ICF (Fig. 3a). This treatment also inhibited shedding of the Dsg2 ectodomain into the cell culture supernatant (Fig. 3b). To identify the specific caspase(s) responsible for Dsg2 ICF generation, IECs were incubated with TNF-α and IFN-γ in the presence of increasing concentrations of selective caspase inhibitors targeting caspases-3/-7 (Z-DEVD-fmk), caspase-8 (Z-IETD-fmk), and caspase-9 (Z-LEHD-fmk), or vehicle. The caspase-8 selective inhibitor, Z-IETD-fmk, had the maximal effect in reducing Dsg2 ICF generation (Fig. 3c). To confirm that caspase-8 is the primary protease responsible for generation of Dsg2 ICF, myc-tag Dsg2 constructs with mutation of two putative intracellular caspase-8 cleavage consensus sites were generated (D715A and D675A). Myc-tagged Dsg2 wild-type (Dsg2 WT) and mutant proteins were expressed in Cos7 cells. Cos7 cells were chosen because they are readily transfected to express exogenous proteins and robustly cleave endogenous and exogenous Dsg2 to generate the Dsg2 ICF. As shown in Fig. 3d, D715A inhibited generation of the Dsg2 ICF while D675A had minimal effects on Dsg2 ICF cleavage in response to TNF-α and IFN-γ treatment. These data indicate that caspase-8 is the primary protease responsible for Dsg2 ICF cleavage following TNF-α and IFN-γ exposure.

### TRAIL promotes Dsg2 cleavage analogous to TNF-α and IFN-γ

**Fig. 4:**
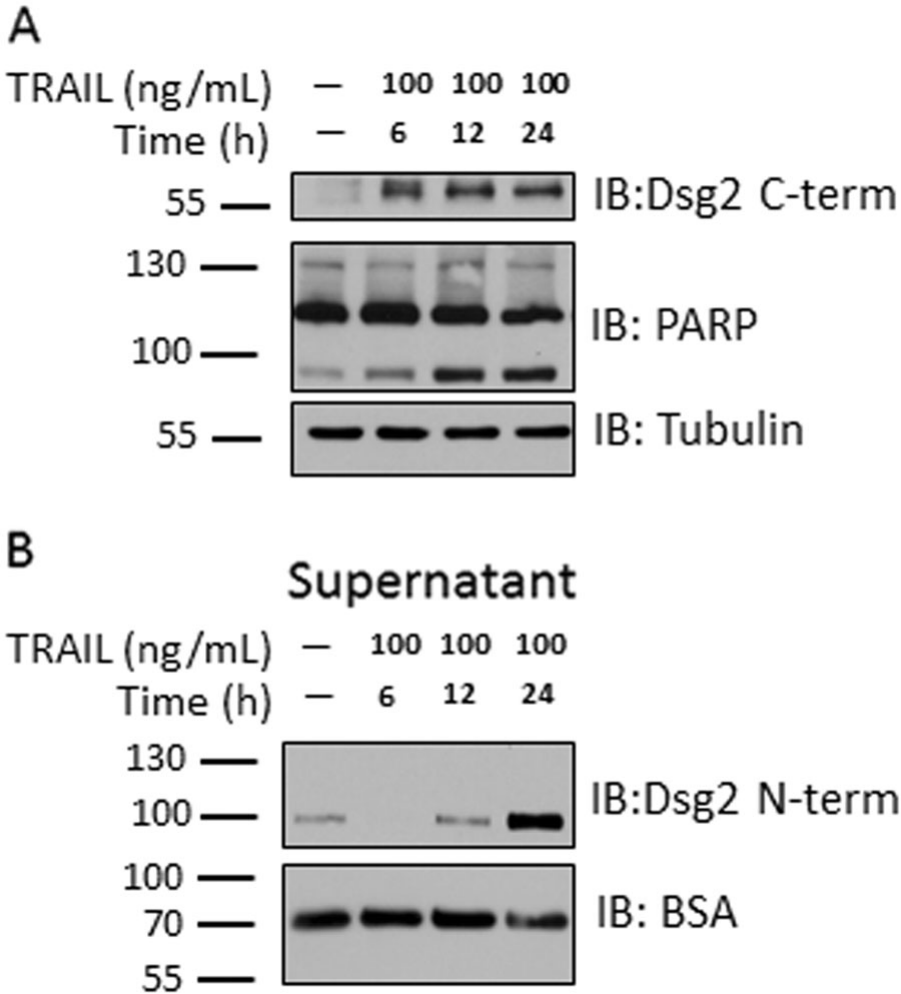
TRAIL treatment induces Dsg2 ECF and ICF generation similarly to TNF-α and IFN-γ. **a** Western blot using an antibody against Dsg2 C-term and against PARP of cell lysates of T-84s treated with TRAIL for 6, 12, or 24 h. **b** Western blot using an antibody against Dsg2 N-term of cell culture supernatants of T-84s treated as in Fig. 4a. All blots are representative of at least three independent experiments

To assess if Dsg2 cleavage can be mediated by another mechanism, which activates the extrinsic apoptotic pathway, a time-course experiment was performed using TNF-α related apoptosis-inducing ligand (TRAIL). TRAIL activates the TNF receptor family members death receptor 4 and death receptor 5 (DR4 and DR5), which initiate the extrinsic apoptosis pathway and caspase-8 activation^26^. As with TNF-α and IFN-γ, Dsg2 ICF generation was induced within the first 6 h of TRAIL treatment (Fig. 4a). Furthermore, TRAIL also induced Dsg2 ectodomain shedding into the cell culture supernatant after initial generation of the Dsg2 ICF (12–24 h) (Fig. 4b). Analogous to TNF-α and IFN-γ treatment, these effects were seen prior to maximal PARP cleavage 12–24 h after TRAIL exposure (Fig. 4a). These data suggest that Dsg2 ICF and ECF generation are a response to stimulation of extrinsic apoptosis.

### Expression of the Dsg2 ICF sensitizes epithelial cells to apoptosis

**Fig. 5:**
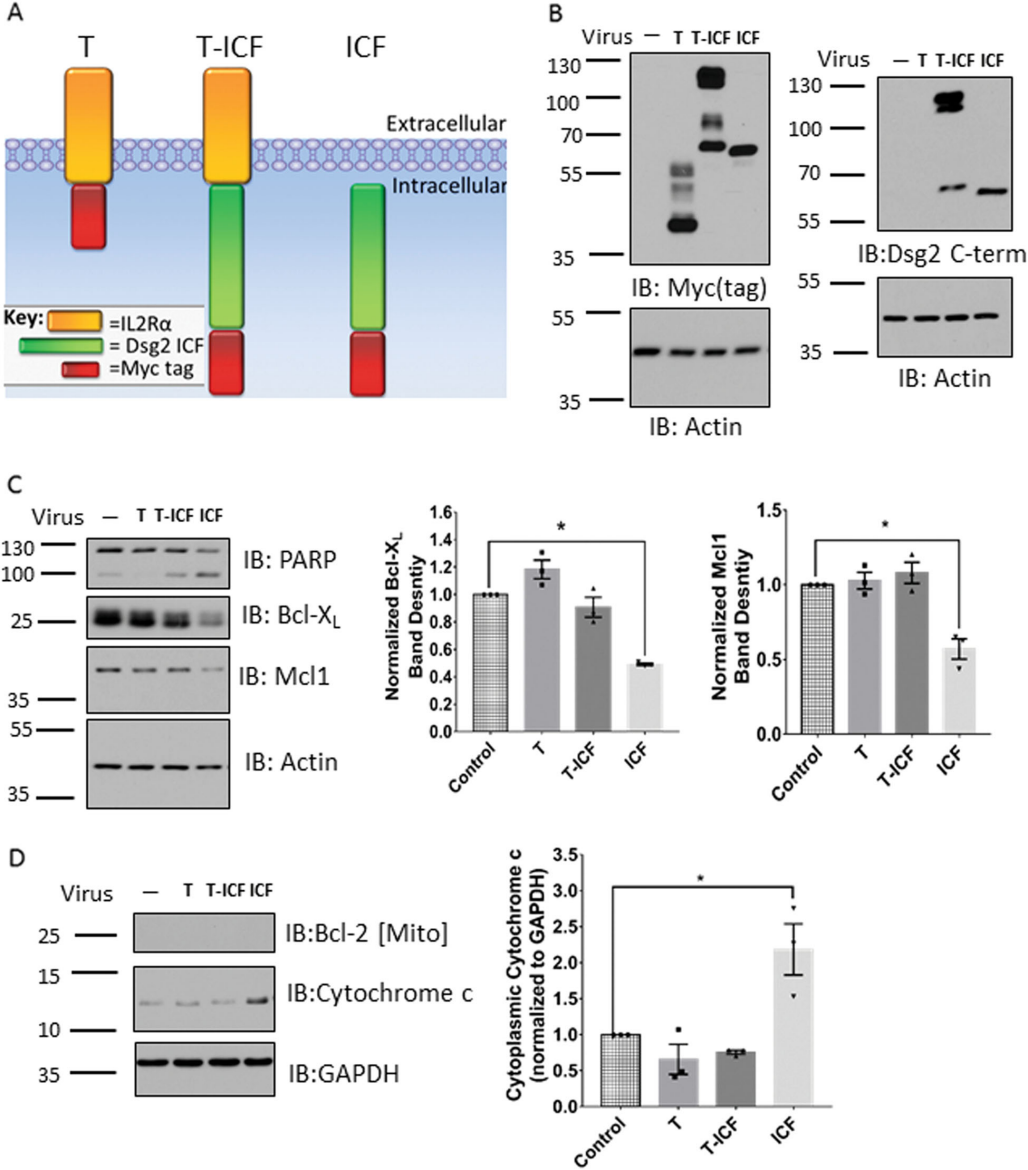
Exogenous expression of Myc-tagged Dsg2 ICF sensitizes cells to apoptosis through downregulation of anti-apoptotic Bcl-2 family members. **a** Model of the protein products of the adenoviral expression constructs used in this paper. **b** Western blot of cell lysates from SKCO15s expressing the indicated adenoviral expression constructs. Left. Western blot for the Myc tag. Right. Western blot using an antibody against Dsg2 C-term. **c** Left. Western blot for PARP, Bcl-X_L_, and Mcl1 of cell lysates from SKCO15s treated as in Fig. 5b. Right. Densitometry of blots for Bcl-X_L_ and Mcl1. *p ≤ 0.05 *n* = 3 per group. For Bcl-X_L_ *p* = 0.0003 and for Mcl1 *p* = 0.0017. **d** Left. Western blot for cytochrome c, GAPDH, and Bcl-2 of cytoplasmic fractions from SKCO15s treated as in Fig. 5b. Right. Densitometry of blots for cytochrome c normalized to GAPDH control. (For similar analysis of mitochondria containing fractions, see Supplemental Fig. 4B.) *p = 0.0093 *n* = 3 per group. All blots are representative of at least three independent experiments. Mito mitochondria, Cyto-c cytochrome c

To determine if Dsg2 ICF generation sensitizes IECs to apoptosis, we exogenously expressed the Dsg2 ICF in SKCO15 model IECs by transduction with an adenovirus-expressing myc-tagged Dsg2 cytoplasmic tail (amino acids 634–1117) (Dsg2 ICF) (Fig. 5a–c). SKCO15 model IECs were used for these experiments since they have well-developed intercellular junctions and can be readily transfected to express proteins. Additionally, to evaluate if plasma membrane tethering influences Dsg2 ICF-mediated signaling and apoptosis, cells were transduced with adenoviruses expressing a fusion construct encoding myc-Dsg2 ICF and the extracellular and transmembrane regions of the IL2R-α receptor (Fig. 5a, b). The IL2R-α plasma membrane tether tagged with myc was expressed as a control (Fig. 5a, b). As shown in Fig. 5b, Dsg2 ICF expression was detected by western blotting using the intracellular-specific epitope-mapped monoclonal Dsg2 antibody 4B2. Analysis of the functional outcome revealed maximal PARP cleavage after expression of the non-tethered Dsg2 ICF-Myc supporting its role in increasing sensitivity to apoptotic stimuli (Fig. 5c). Cells expressing the non-tethered Dsg2 ICF also displayed a decrease in the protein levels of the anti-apoptotic Bcl-2 family members Bcl-X_L_ and Mcl1 (~49 and ~57% of control, respectively) (Fig. 5c). To assay the effects of Dsg2 ICF expression on mitochondrial outer membrane permeabilization and cytochrome c release into the cytoplasm, we harvested cytoplasmic and mitochondria containing fractions of model IECs transduced with the above adenovirus constructs. Fractionation controls are shown in Supplemental Fig. 4a. The cytoplasmic cytochrome c levels were increased in cells expressing the non-tethered Dsg2 ICF compared to controls (~218% of control) (Fig. 5d). To further confirm increased apoptosis in cells expressing the Dsg2 ICF, Annexin V labeling was performed in live cells. Cells expressing the Dsg2 ICF displayed increased Annexin V labeling and cell loss compared to controls (Supplemental Fig. 5). These findings suggest that cleavage and release of Dsg2 ICF from the plasma membrane is associated with sensitization to apoptotic signaling and that downregulation of anti-apoptotic Bcl-2 family members contributes to this process.

## Discussion

Mechanisms of epithelial barrier compromise in response to inflammation remain incompletely understood. Mucosal inflammation perturbs IEC homeostatic signaling that is associated with compromised barrier function, which results in translocation of luminal antigen that further perpetuate the inflammatory response. Failure to control inflammation and epithelial barrier dysfunction has been observed in chronic diseases such as inflammatory bowel disease (IBD)^2,27^. Thus, understanding the mechanisms of epithelial barrier compromise is important in developing effective therapeutic strategies. Homeostatic mechanisms that are perturbed during mucosal inflammation include proteolytic cleavage of transmembrane cadherin proteins resulting in the generation of functional protein fragments^5,8,11,13,15,22,23,28–32^. Few studies describe cadherin cleavage specifically in response to inflammatory stimuli. These primarily focus on the generation of extracellular cadherin fragments, which usually interfere with cadherin-based adhesion or the maintenance of endothelial barrier tightness^5,31,32^. Regardless of the stimulus responsible, in most scenarios the extracellular cadherin cleavage precedes intracellular cleavage. These sequence of events have been described for E-cadherin, N-cadherin, and VE-cadherin as well as Dsg2 and Dsg1 in keratinocytes^15,22,23,29,30^. In contrast to these reports, we observed that Dsg2 undergoes intracellular cleavage prior to extracellular cleavage in response to pro-inflammatory cytokine signaling in intestinal epithelial cells. These differences are likely related to the cell type and stimulus that induces such responses. Little is known about the relationship between intracellular and extracellular cadherin cleavage in response to inflammatory stimuli. However, one report showed that upon exposure of HUVECs to TNF-α, VE-cadherin undergoes cleavage in its extracellular and intracellular domains, and inhibiting extracellular cleavage suppresses intracellular cleavage^30^. This suggests that, in response to TNF-α, VE-cadherin extracellular cleavage occurs prior to its intracellular cleavage.

Knowledge of the protease(s) responsible for generation of protein fragments provides crucial context to the functional effects of that fragment within the cell and in the overall pathology. Cadherin extracellular cleavage is most often mediated by matrix metalloproteinases. ADAM10 promotes extracellular cleavage of E-, N-, and VE-cadherin resulting in the generation of N-terminal fragments (NTFs). In a recent report, we observed that ADAM10 and MMP9 were responsible for extracellular cleavage of Dsg2 in intestinal epithelial cells^5,11,28,29^. In keratinocytes, ADAM 17 has been implicated in mediating extracellular cleavage of Dsg2^15^.

Cadherin intracellular cleavage, however, can be somewhat complicated and several protease families have been implicated in mediating this event. For example, the most abundant E-cadherin C-terminal fragment (CTF2) is generated by γ-secretase, but other reports implicate cysteine proteases in mediating E-cadherin intracellular cleavage^22,24,33^. Surprisingly, despite both the mechanistic similarities to notch signaling (extracellular cleavage by ADAM10 and generation of a functional intracellular fragment) as well as the similarities of the Dsg2 ICF to the CTF2s of both E-cadherin and N-cadherin, we found that γ-secretase inhibition had no effect on Dsg2 ICF generation^22,23,34,35^. We therefore investigated the influence of other protease families in mediating Dsg2 intracellular cleavage.

The calpain family of intracellular cysteine proteases have been shown to mediate E-cadherin intracellular processing^24^. Such E-cadherin intracellular cleavage was shown to leave behind a membrane-bound ~100 kDa N-terminal fragment^24^. Thus, analogous to the processing of Dsg2 in IECs, calpain-mediated E-cadherin cleavage appears to occur before extracellular cleavage. However, we observed that inhibition of calpains caused an increase in Dsg2 ICF generation indicating that the activity of the protease responsible for generating this fragment was enhanced by calpain inhibition. We also observed that calpain inhibition resulted in increased abundance of active caspase-8. Not only have members of the caspase family of cysteine proteases been implicated in cadherin cleavage, their activity is known to be regulated by the calpains^14,22,25^. While it is generally thought that calpains cleave and activate caspase-3, a previous report has shown that calpains cleave and inactivate the initiator caspases, caspase-8 and caspase-9^25,36–38^. In keratinocytes, intracellular Dsg1 cleavage is mediated by caspases-3/-7. The cleavage site was identified to reside within the third Dsg1 repeated unit domain (RUD) thereby generating a ~17 kDa fragment^14^. Cirillo et al. previously attributed generation of the Dsg2 ICF to caspase-3 based upon inhibitor studies using the caspase-3 selective inhibitor Z-DEVD-fmk^39^. In our studies, Dsg2 ICF generation by TNF-α and IFN-γ was minimally influenced by this inhibitor at a concentration of 30 μM. However, using 100 μM Z-DEVD-fmk inhibited Dsg2 intracellular cleavage. It is to be cautioned that at high doses such inhibitors often have off-target effects^40^. Our study suggests a more definitive role of caspase-8 in mediating Dsg2 intracellular cleavage. These results were supported by performing dose–response studies using selective caspase inhibitors as well as by expression of two Dsg2 mutant constructs lacking putative caspase-8 cleavage sites (D715A and D675A). These sites were chosen based upon their ability to generate a ~55 kDa ICF and their similarities to known caspase-8 cleavage consensus sites. The identification of caspases as mediators of cadherin intracellular cleavage suggests that these mechanisms may play a larger role in apoptosis regulation.

The two primary pathways that lead to the execution of apoptosis are the extrinsic and intrinsic pathways. Caspase-8 is one of the initiator caspases for the extrinsic pathway. This pathway is closely related to TNF-α pro-inflammatory signaling^17^. This is because TNFR1 is one of the founding members of the “death receptor” family of transmembrane receptors whose activation is the first step in the extrinsic pathway^41^. TRAIL is another canonical extrinsic apoptotic stimulus that activates death receptors 4 and 5 (DR4/5)^42^. In this study, we found that the Dsg2 ICF is generated in response to TRAIL treatment with similar kinetics to TNF-α and IFN-γ treatment. These observations indicate that generation of the Dsg2 ICF is a response to stimulation of the extrinsic apoptotic pathway. Thus, Dsg2 ICF generation may play a role in the response to extrinsic apoptosis activation through inflammatory mediators and in other scenarios such as use of chemotherapy agents that target DR4/5 or CD95^43,44^. Following stimulation of death receptors, the extrinsic apoptotic cascade proceeds through activation of caspases-8 and/or -10. Once these initiator caspases have been activated, they cleave target proteins including pro-caspase-3 leading to its activation and the execution of apoptosis^17^. Due to the irreversible nature of activation through proteolysis, caspase-3 activation and apoptosis execution are tightly regulated through numerous upstream sensitization pathways. Therefore, the generation of the Dsg2 ICF by caspase-8 in response to both TNF-α and IFN-γ as well as TRAIL suggests that Dsg2 cleavage can represent a potential mechanism of sensitization in the apoptotic cascade. Since the sensitivity of IECs to apoptosis is a key factor in whether or not mucosal inflammation progresses toward effective resolution or development of chronic disease, this mechanism may play a role in the pathobiology of epithelial barrier dysfunction in inflammatory diseases.

There is a precedent for cadherin intracellular cleavage playing a role in apoptotic mechanisms. Most reports describing cadherin intracellular cleavage events used apoptotic stimuli. Although most of these have been intrinsic stimuli such as UV exposure, staurosporine, or camptothecin^14,22,33^. However, the generation of the VE-cadherin intracellular fragment in response to TNF-α has been described^29^. The same report that identified Dsg1 intracellular cleavage by caspases-3/-7 also showed that keratinocytes displayed increased resistance to UV-induced apoptosis in response to shRNA knockdown of Dsg1^14^. We have obtained similar results previously using siRNA knockdown of Dsg2 in IECs treated with camptothecin^13^. In the current study, we observed that expression of Myc-tagged Dsg2 ICF in model IECs leads to increased sensitivity to apoptosis. IECs expressing the Dsg2 ICF also displayed reduced levels of the anti-apoptotic Bcl-2 family proteins, Bcl-X_L_ and Mcl1, at the protein level. The Bcl-2 family of proteins are key regulators of mitochondrial engagement in apoptosis, which is essential for intrinsic apoptosis as well as extrinsic apoptosis in type II extrinsic apoptotic cells such as IECs^18^. These data indicate that the Dsg2 ICF does, in fact, participate in sensitization to apoptotic stimuli and suggest that downregulation of Bcl-X_L_ and Mcl1 plays a role in this process.

**Fig. 6:**
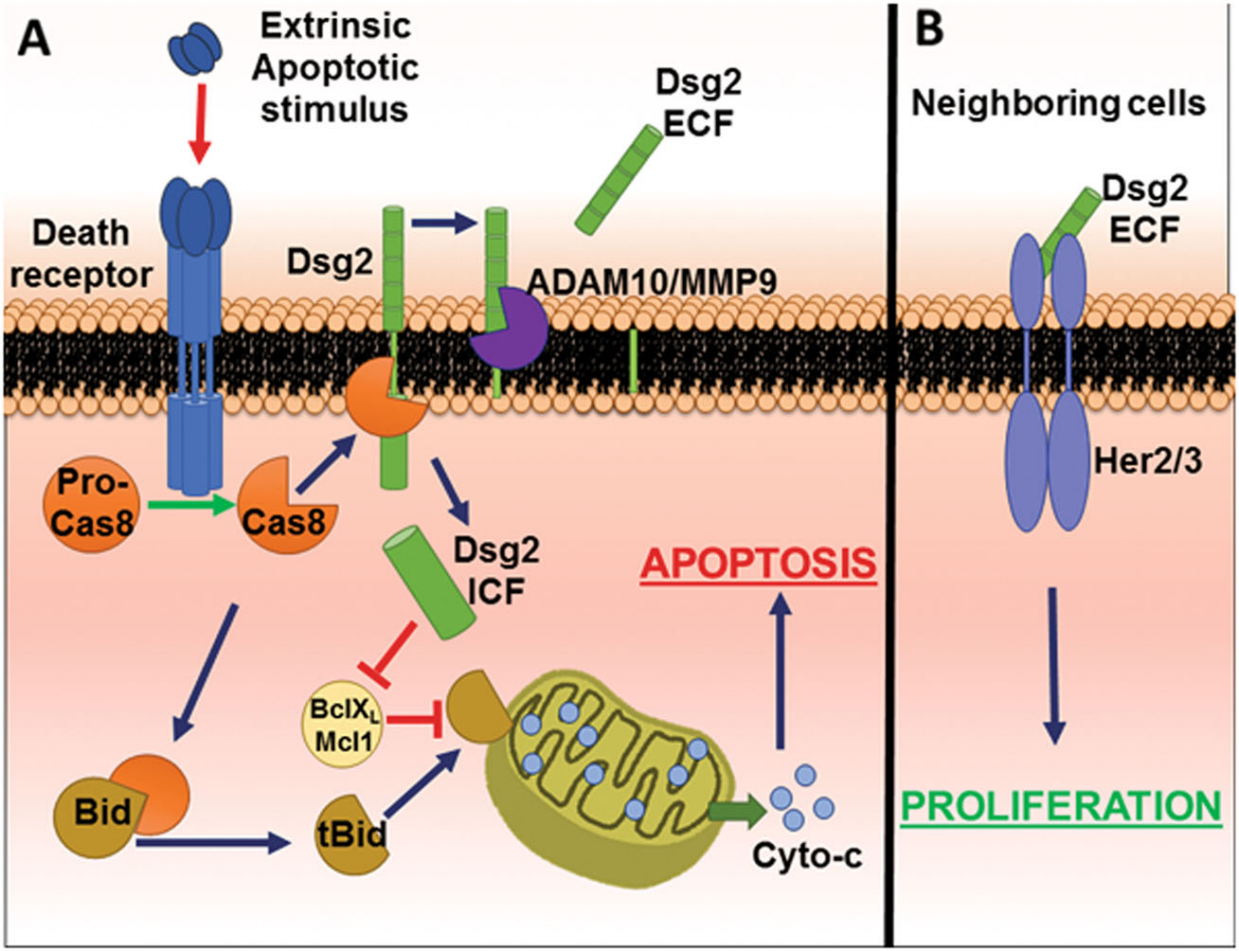
Dsg2 ICF generation sensitizes IECs to apoptosis. **a** Stimulation of death receptors (such as TNFR1 or DR4/5) results in activation of caspase-8, which cleaves Dsg2 intracellularly generating the Dsg2 ICF as well as other substrates including the Bcl-2 family member Bid-generating tBid. The presence of the Dsg2 ICF reduces Bcl-X_L_ and Mcl1 protein levels, thereby alleviating their inhibition of tBid and allowing for MOMP and the execution of apoptosis to proceed. Following Dsg2 ICF generation, MMP9 and ADAM10 cleave Dsg2 in the extracellular domain generating the Dsg2 ECF. **b)** We have previously shown that this Dsg2 ECF promotes wound closure by stimulating IEC proliferation through activation of Her2/3 signaling. We propose that the Dsg2 ICF and ECF work in concert to remove damaged IECs and promote wound closure to help ensure effective resolution of inflammation. Pro-Cas8 Pro-caspase-8, Cas8 caspase-8, Cyto-c, cytochrome c

We have shown that TNF-α and IFN-γ exposure induces cleavage of Dsg2 mediated by caspase-8 leading to the generation of the Dsg2 ICF. Presence of this Dsg2 ICF causes sensitization to apoptosis through downregulation of the anti-apoptotic Bcl-2 family members Bcl-X_L_ and Mcl1. This establishes Dsg2 intracellular cleavage as a mechanism that mediates apoptosis of cells that are exposed to an inflammatory insult (Fig. 6a). Of interest, the dying cells subsequently release the Dsg2 ECF, which can signal to surrounding cells to promote proliferation and repair through activation of HER2 and HER3 signaling (Fig. 6b)^5^. Considering that the Dsg2 ICF is generated before the Dsg2 ECF, we propose that during mucosal inflammation these events mediate death and loss of damaged IECs through Dsg2 ICF signaling, while subsequent generation of the Dsg2 ECF can have paracrine effects on surrounding epithelial cells to promote proliferation and repair.

## Materials and methods

### Antibodies and reagents

The following primary monoclonal and polyclonal antibodies were used to detect proteins by immunoblot analysis: mouse anti-Dsg2 {extracellular} (clone AH12.2, generated in-house^13,45^ [1:1000], mouse anti-Dsg2 {intracellular} [1:750] (clone 4B2; a kind gift from Dr. K.J. Green, Northwestern University, Evanston, IL), mouse anti-beta-actin (clone AC-74) [1:5000-1:10,000], rabbit anti-glyceraldehyde 3-phosphate dehydrogenase (GAPDH) [1:20,000], mouse anti-tubulin (clone DM1A [1:10,000]; Sigma-Aldrich; St. Louis, MO, USA), rabbit anti-cleaved PARP (clone D64E10 XP(r)) [1:1000], rabbit anti-PARP [1:1000], rabbit anti-Bcl-X_L_ (clone 54H6) [1:2000], rabbit anti-cleaved Notch (clone D3B8) [1:1000], rabbit anti-cytochrome c (clone D18C7) [1:1000], mouse anti-caspase-8 (clone 1C12), mouse anti-Myc tag (clone 9B11; Cell Signaling Technology; Danvers, CT, USA) [1:5000], rabbit anti-Na/K ATPase [1:100,000] (clone EP1845Y; Abcam; Cambridge, MA, USA), rabbit anti-Mcl1 [1:3000] (Enzo Life Sciences; Farmingdale, NY, USA), Armenian hamster anti-Bcl-2 [1:125] (BD Biosceicnes; San Jose, CA, USA), rabbit anti-bovine serum albumin (BSA) (clone B-140) [1:10,000], rat anti-guanylate binding protein-1 (GBP-1 (clone 1B1) [1:750]; Santa Cruz Biotechnology, Santa Cruz, CA, USA). Goat anti-mouse and anti-rabbit HRP-conjugated secondary antibodies were obtained from Jackson ImmunoResearch Laboratories (West Grove, PA, USA) and used at a concentration of 1:10,000. Mouse anti-armenian/Syrian hamster HRP-conjugated secondary antibody cocktail (clones G70-204/G94-56) was purchased from BD Biosciences and used at a concentration of 1:1000. Recombinant human TNF-α, IFN-γ, and TRAIL were purchased from PeproTech (Rocky Hill, NJ, USA). Z-VAD-fmk was purchased from Enzo Life Sciences. Calpeptin was purchased from EMD Millipore (Billerica, MA, USA), GI454023X was purchased from Sigma-Aldrich, Z-DEVD-fmk, Z-LEHD-fmk, and Z-IETD-fmk were all purchased from R&D Systems (Minneapolis, MN, USA).

### Cell culture

T-84 and SKCO15 human model IEC were cultured both on Transwell™ permeable supports and on tissue culture treated plastic as previously described^46,47^. Cos7 monkey model kidney fibroblast cells were cultured as described previously^48^. For TNF-α+IFN-γ dose dependency experiments, cells were treated with media alone (control), 100 Units/mL of IFN-γ+10 ng/mL TNF-α, or 200 Units/mL of IFN-γ+20 ng/mL TNF-α. For time-course experiments, T-84 model IECs were treated with either 200 Units/mL of IFN-γ+20 ng/mL TNF-α or 100 ng/mL of TRAIL for 0, 6, 12, and 24 h. For transient plasmid transfection, Lipofectamine 3000 (Invitrogen; Carlsbad, CA, USA) was used according to the manufacturer’s instructions. Cell culture media was replaced 24 h after transfection with either media alone or media containing 200 Units/mL of IFN-γ+20 ng/mL TNF-α. Samples were collected for immunoblotting 24 h after application of cytokine or control treatment. For cells cultured on Transwell permeable supports with 3 μm pore size filters (Corning), transepilthelial electrical resistance was measured using an EVOM voltmeter with an ENDOHM-12 (World Precision Instruments; Sarasota, FL, USA). Electrical resistance was expressed as Ωxcm^2^. For calculation, the resistance of blank filters was subtracted from that of filters covered with cells.

### Inhibitor treatment

Confluent T-84 cells on Transwell permeable supports were treated with the following inhibitors 1 h before treatment with 200 Units/mL of IFN-γ+20 ng/mL TNF-α: 10 μM GI254023X, 50 μM Z-VAD-fmk, 20/50 μM calpeptin, 10 μM DAPT, 10 μM/30 μM/100 μM Z-DEVD-fmk, 10 μM/30 μM/100 μM Z-IETD-fmk, 10 μM/30 μM/100 μM Z-LEHD-fmk.

### Immunoblotting

For in vitro cell cultures, confluent monolayers were collected in RIPA (20 mM Tris-Base, 150 mM NaCl, 2 mM EDTA, 2 mM EGTA, 1% sodium deoxycholate, 1% Triton X-100, 0.1% SDS, pH 7.4) containing protease and phosphatase inhibitor cocktails (Sigma-Aldrich). Cells were incubated at 4 °C for 20 min and centrifuged (8000 × *g* at 4 °C for 10 min). For cell culture supernatants, supernatants were cleared by centrifugation (5000 × *g* at 4 °C for 5 min). Protein concentration was determined using a Pierce BCA Protein Assay Kit (Thermo Fisher Scientific; Marietta, OH, USA). NuPAGE LDS Sample Buffer (Life Technologies; Eugene, OR, USA) with a final concentration of 100 mM DTT (Sigma-Aldrich) was used. Samples were boiled for 5 min at 100 °C and loaded onto polyacrylamide gels (TNFα+IFN-γ or TRAIL lysate samples = 30 μg protein/lane; adenoviral transduction lysates = 10 μg protein/lane; cell culture supernatants = 30 μL of sample/lane; cell fraction samples = 22 μg protein/lane). After electrophoresis, the samples were transferred to a nitrocellulose membrane (Bio-Rad; Hercules, CA, USA) and probed with primary antibodies and HRP-conjugated secondary antibodies (Jackson ImmunoResearch Labs). Immunoblots were quantified using ImageJ (National Institutes of Health; Bethesda, MD, USA) or an Amersham 600 Blot Imaging System (GE Healthcare; Piscataway, NJ, USA). To calculate the amount of the Dsg2 ICF generated under each condition in Fig. 1b, f, the density of bands representing Dsg2 cleavage products as well as the full-length protein were collected and the ratio of the density of the Dsg2 ICF band to the density of all bands for a given lane was then calculated. For all densitometric analysis, each individual data point was derived from measurement of a distinct sample.

### Adenovirus transduction

Fifteen minutes prior to adenoviral transduction, SKCO15 model IECs were calcium switched using calcium switch reagent (10 mM HEPES, 25 mM EGTA, pH 7.4). Following this, the Dsg2 ICF-Myc and IL2R-Dsg2 ICF-Myc viruses were added at an MOI of 1 and the IL2R-Myc virus was added at an MOI of 10 to ensure equal exogenous protein expression. Cells were centrifuged for 1 h at 1200 × *g* at 37 °C and then placed into a humidified incubator with 5% CO_2_ at 37 °C. Samples were used 48 h following initial virus addition unless otherwise specified.

For western blot analysis, sample preparation was as described above.

### Cell fractionation

To generate the cytoplasmic and mitochondria containing cellular fractions, we used the Compartmental Protein Extraction Kit (Millipore; Temecula, CA, USA) according to the manufacturer’s instructions.

### Immunofluorescence labeling

Confluent T-84 monolayers grown on Transwell permeable supports were fixed using 4% paraformaldehyde in PBS+ for 15 min. Samples were washed three times using PBS+ and then permebialized using 0.5% Triton X-100 in PBS+ for 10 min. Following washing, samples were blocked in 3% BSA in PBS+ for 30 min and labeled with 4B2 [1:100] in 3% BSA in PBS+ for 2 h. The samples were then washed and incubated with goat anti-mouse secondary antibodies conjugated with Alexa Fluor 488 (Invitrogen; [1:1000]) in 3% BSA in PBS+ for 1 h, washed and mounted onto charged glass slides using Prolong gold (Life Technologies; Eugene, OR, USA). Images were collected on a Leica SP5 confocal microscope and processed using ImageJ.

### Annexin V fluorescent imaging

Alexa Fluor 568-conjugated Annexin V (Life Technologies) was used for fluorescent live-cell imaging according to the manufacturer’s instructions with minor modifications. Briefly, model SKCO15 cells were transduced with the Dsg2 ICF-Myc, IL2R-Dsg2 ICF-Myc, and IL2R-Myc adenoviral constructs as described above. Cells were washed in ice-cold PBS+ three times followed by incubation with 10 μL of labeled Annexin V per 100 μL of annexin-binding buffer (10 mM HEPES, 140 mM NaCl, 2.5 mM CaCl_2_, pH 7.4) for 15 min. Cells were washed three times using annexin-binding buffer and imaged using a Zeiss Axiovert 200M microscope. Images were exported as TIFFs and processed using ImageJ.

### Statistics

Statistical significance was calculated using either one-way ANOVA followed by Dunnet’s test for multiple comparisons or two-way ANOVA followed by Tukey’s test for multiple comparisons dependent upon the experimental design. Results displayed as means ± SEM; *p* < 0.05 was considered significant.

## Electronic supplementary material

Supplemental Figure Legends

Supplemental Figure 1

Supplemental Figure 2

Supplemental Figure 3

Supplemetnal Figure 4

Supplemental Figure 5
