# Supplementary material for: Intracellular Desmoglein-2 cleavage sensitizes epithelial cells to apoptosis in response to pro-inflammatory cytokines
Source: Cell Death Dis. 2018 Mar 9;9(3):389. doi: 10.1038/s41419-018-0380-9 (PMC5844960; doi:10.1038/s41419-018-0380-9)
Supplement: Supplementary file 1 — Supplemental Figure Legends [file 41419_2018_380_MOESM1_ESM.docx]

Supplemental Figure 1: **A)** Western blot for GBP-1 and PARP of cell lysates of T-84s treated with TNF-α and IFN-γ for 24 h. All blots are representative of at least three independent experiments. **B)** TEER of cells used in Figure S1A and in Figure 1. Treatment was applied on day 6 post plating as indicated by the black arrow. TEER measurements were collected as described in *Materials and Methods.* *, p=0.0001 n=11 per group.

Supplemental Figure 2: **A)** Full lanes of western blot using an antibody against Dsg2 C-term shown in Figure 1B. **B)** Full lanes of western blot using an antibody against Dsg2 N-term shown in Figure 1C. All blots are representative of at least three independent experiments.

Supplemental Figure 3: Immunofluorescence staining and confocal microscopy using an antibody against Dsg2 C-term in T-84 cells treated as in Figure 1B. Scalebars=20 μm. Images are representative of at least three independent experiments.

Supplemental Figure 4: **A)** *Top.* Western blot for Na/K ATPase, PARP, and GAPDH from indicated fractions of SKCO15 cells treated as in Figure 5B [Control and T virus transduced samples]. *Bottom.*  Western blot for Na/K ATPase, PARP, and GAPDH from indicated fractions of SKCO15 cells treated as in Figure 5B [T-ICF and ICF virus transduced samples]. All blots are representative of at least three independent experiments. We have found that the mitochondria are enriched in the membrane fraction and are absent from the cytoplasmic fraction when using this fractionation kit (Milipore; Tamecula, CA, USA). Abbreviations: Cyto, cytoplasmic; Mem, membrane (mitochondria containing); Nuc, nuclear. **B)** Western blot for cytochrome c, Na/K ATPase, and Bcl-2 of mitochondria containing fractions from SKCO15s treated as in Figure 5B.

Supplemental Figure 5: Fluorescence and light microscopy merged images of SKCO15 cells treated as in Figure 5B and labeled with Alexa fluor 568 conjugated annexin V [red]. GFP is in green. All images are representative of at least three independent experiments. Scalebars=100 μm. Images are representative of at least three independent experiments.
