# Supplementary figures and images for: Intracellular Desmoglein-2 cleavage sensitizes epithelial cells to apoptosis in response to pro-inflammatory cytokines
Source: Cell Death Dis. 2018 Mar 9;9(3):389. doi: 10.1038/s41419-018-0380-9 (PMC5844960; doi:10.1038/s41419-018-0380-9)

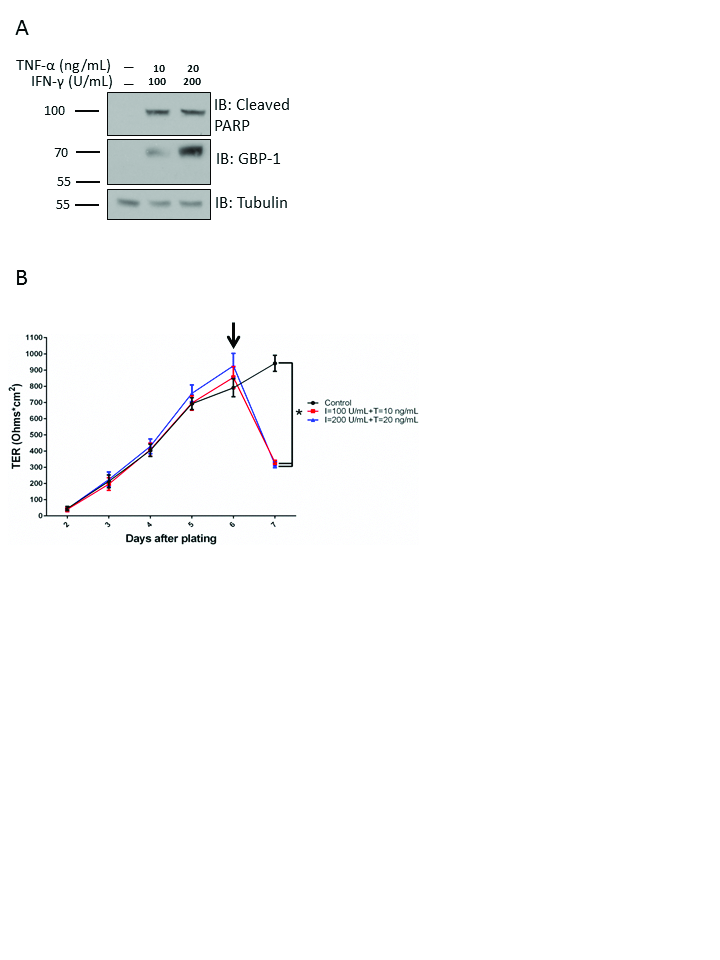

Supplement: Supplementary file 2 — Supplemental Figure 1 [file 41419_2018_380_MOESM2_ESM.tif]

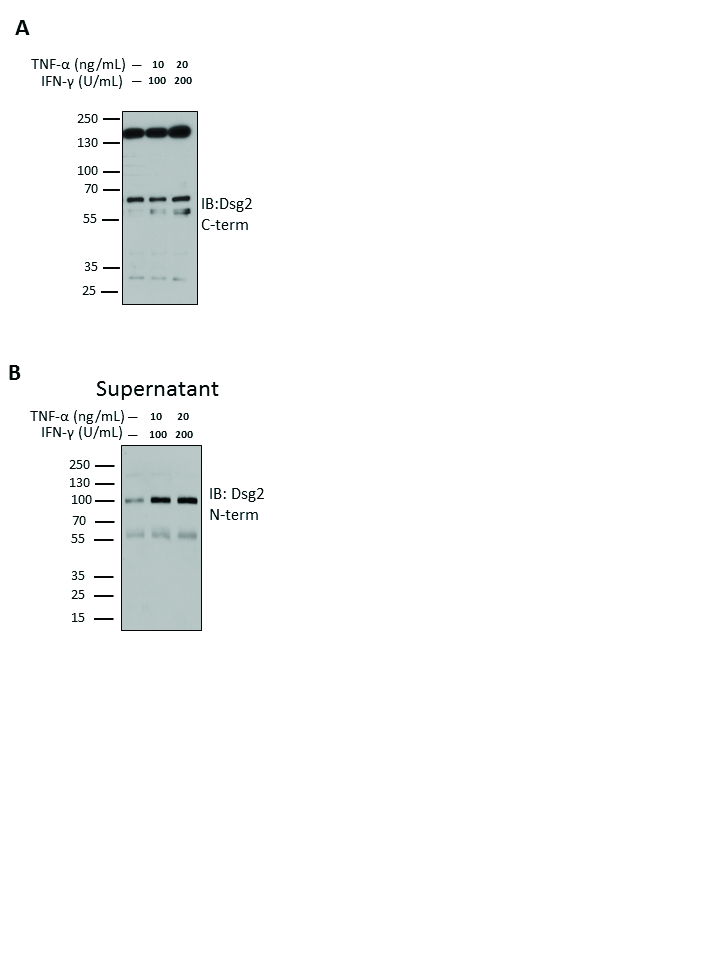

Supplement: Supplementary file 3 — Supplemental Figure 2 [file 41419_2018_380_MOESM3_ESM.tif]

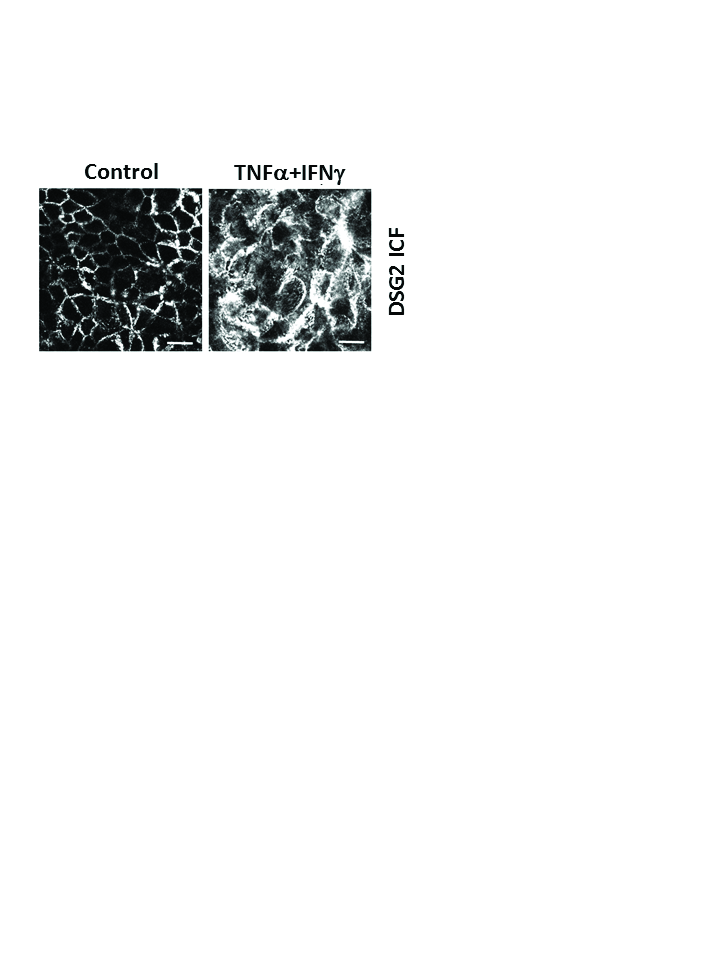

Supplement: Supplementary file 4 — Supplemental Figure 3 [file 41419_2018_380_MOESM4_ESM.tif]

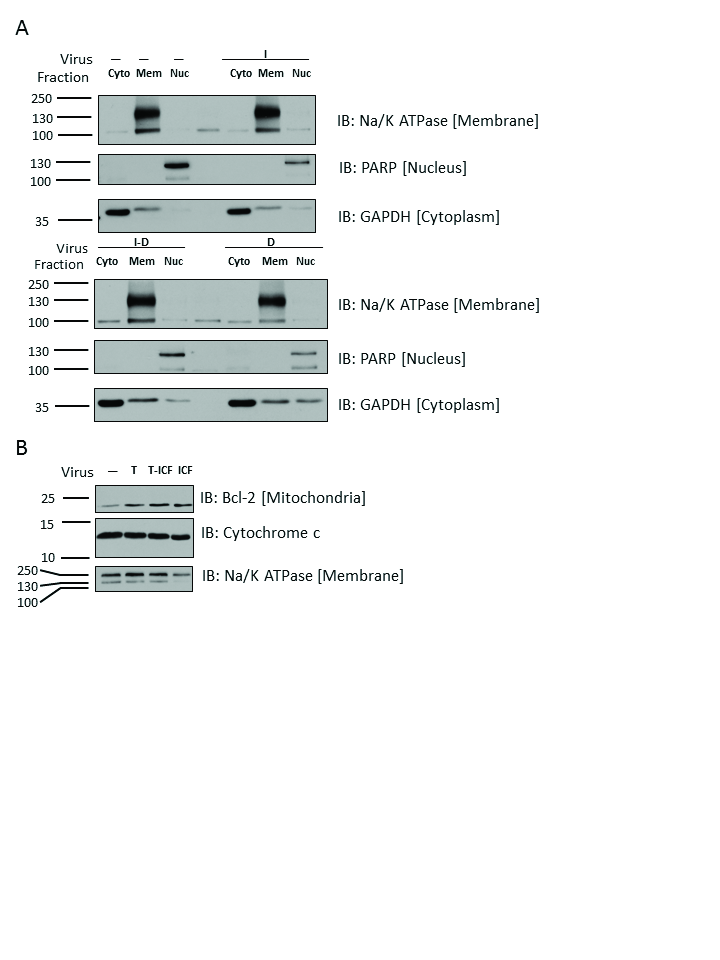

Supplement: Supplementary file 5 — Supplemetnal Figure 4 [file 41419_2018_380_MOESM5_ESM.tif]

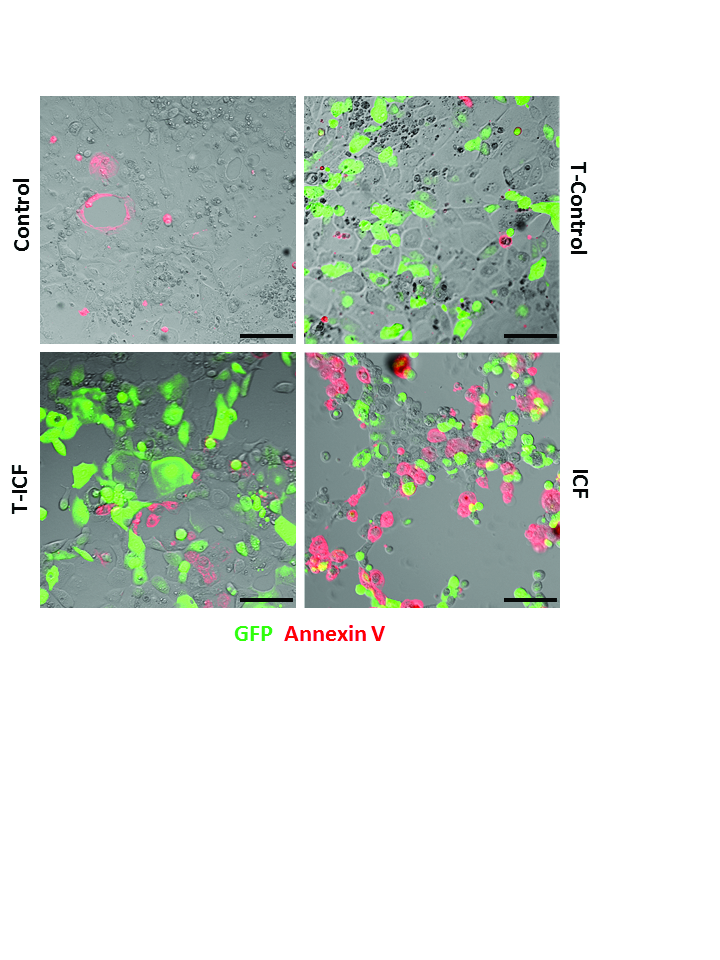

Supplement: Supplementary file 6 — Supplemental Figure 5 [file 41419_2018_380_MOESM6_ESM.tif]
